# The Anatomy of the Arteries of the Human Body

**Published:** 1841-07-01

**Authors:** 


THE ANATOMY OF THE ARTERIES OF THE HUMAN BODY, WITH ITS APPLICATIONS TO PATHOLOGY, AND OPERATIVE SURGERY, IN LITHOGRAPHIC DRAWINGS, WITH PRACTICAL COMMENTARIES. By *Richard Quain*, Professor of Anatomy in University College, and Surgeon to University College Hospital. The Delineations by *Joseph Maclise*, Esq. Surgeon. Parts 1 to 6. Price 12s. each. London: Taylor and Walton.

It has been a subject of painful regret to ourselves that no sufficient notice has hitherto been taken in this Journal of the splendid work before us. It reflects high credit, not on the author only but this country, and is, in every way, worthy of both.

The author, in his advertisement, thus acquaints us with the aim and with the plan of the work.

The object, he says, of this publication is to lay before the student and practioner by means of accurate delineations, and a record of the peculiarities observed in numerous cases, a correct history of the blood-vessels, arranged especially with a view to its bearing on practical surgery.

Mr. Quain informs us in his Preface, that,—

"Several years have elapsed since I became impressed with the belief that the difficulties which have often occurred in the performance of those surgical operations in which the larger arteries are concerned, have arisen in great part from want of sufficient acquaintance with the differences in anatomical disposition to which these vessels are subject—not merely those deviations in the origin of large branches, which are usually named varieties, but other peculiarities of various kinds which are liable to occur, such as those which affect the length, position, or direction of the vessels. Under that impression I was led to observe these circumstances more closely, and finally determined to obtain a record of the condition, whatevei it might be, of the more important vessels in a considerable number of cases,—a record to be made especially with a view to points bearing on practical surgery.

With this view, I examined with more or less attention the bodies which were received during a series of years for the study of anatomy into the School of Medicine in University College. These bodies, to the number of 930, were with rare exceptions so inspected with reference to the subject of my inquiries, that anything very unusual could not escape notice: and, in order to insure accuracy, when other occupations allowed, the arteries were carefully examined and their condition noted at the time, attention being always particularly directed to those vessels and to the points in their history which seemed to be of importance in the practice of surgery.

This detailed investigation was continued until the number of cases observed appeared such as would afford grounds for reasonable conclusions both as to the limits of the deviations from the ordinary standard, and as to the relative frequency of their occurrence.

At the same time that the observations thus made were written down, drawings were obtained of all the important peculiarities which presented themselves, and when it was practicable the preparations were preserved.

The varieties in the arrangement of the blood-vessels thus noted grew, as may be supposed, to be very numerous; but instead of difficulties multiplying with the number of observations, it was usually found that as the facts accumulated, the transition from one state to a very different one ceased to be abrupt or without method, for others from time to time interposed which served to link them together.

Originally these observations were intended exclusively for the benefit of my class; but as their number and connexion seemed likely to render them more extensively useful, I resolved to publish them. On examining with a view to publication the materials which I had collected, it became obvious that their utility would be very limited, unless as a part of a full history of the arteries with adequate delineations. In consequence, a series of drawings, showing the arteries according to their usual arrangement, has been prepared, and to these are appended the observations previously alluded to. The work has thus grown under my hands, and has gradually assumed its present form." 6.

The Parts before us are admirable. The sketches are clear, forcible, and to the life. Every operating surgeon should possess them—every professional library and reading room should contain them. It is chiefly by the patronage of Institutions that Mr. Quain can be remunerated, and it would be disgraceful if he were not.

